# General corrosion during metal-assisted etching of *n*-type silicon using different metal catalysts of silver, gold, and platinum

**DOI:** 10.1039/c9ra08728a

**Published:** 2020-01-02

**Authors:** Ayumu Matsumoto, Hikoyoshi Son, Makiho Eguchi, Keishi Iwamoto, Yuki Shimada, Kyohei Furukawa, Shinji Yae

**Affiliations:** Department of Chemical Engineering and Materials Science, Graduate School of Engineering, University of Hyogo 2167 Shosha Himeji Hyogo 671-2280 Japan matsumoto.ayumu@eng.u-hyogo.ac.jp

## Abstract

Metal-assisted etching is a promising technique for microfabrication of semiconductors. In this method, porous silicon (Si) can be produced with a very simple procedure, and various nanostructures can be designed by changing the catalyst patterns. The kind of metal catalysts is one of the key factors to control the porous structure. In this work, we performed the etching of *n*-type Si (100) in a hydrofluoric acid solution containing hydrogen peroxide in the dark using silver, gold, and platinum particles electrolessly deposited at a constant coverage, and demonstrated the difference in the porous structures obtained for the different kind of metal catalysts. By comparing the mass loss of substrates with the depth of pores formed under the metal particles, we found that general corrosion occurred on the top-surface of the Si substrate around the metal particles even under the dark condition. The general corrosion depended on the metal species and it was explained by the formation and dissolution of a mesoporous layer. The kind of metal catalysts influences the dissolution of the Si surface not only under the metal catalysts but also between them.

## Abstract 2

The first report on general corrosion during metal-assisted etching of silicon.

## Graphical abstract

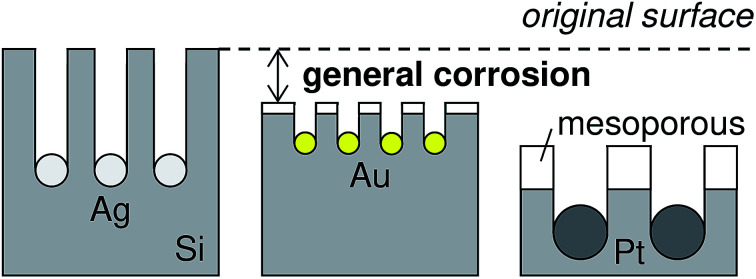

## 1. Introduction

Metal-assisted etching (or metal-assisted chemical etching (MacEtch)) has attracted keen attention as a cost-effective and versatile microfabrication technique for semiconductors.^1,2^ In this method, porous silicon (Si) can be produced with a very simple procedure in which a Si substrate modified with metal catalysts is immersed in a hydrofluoric acid (HF) solution containing an oxidizing agent, such as hydrogen peroxide (H_2_O_2_),^3,4^ dissolved oxygen (O_2_),^5^ and metal ions.^6^ Under optimized conditions, the Si surface under the metal catalysts is selectively etched, and therefore, we can fabricate various nanostructures by changing the catalyst patterns.

As examples of the applications, Si nanohole arrays and Si nanowires produced by the metal-assisted etching have been applied to anti-reflection for solar cells,^5,7,8^ anode materials of lithium-ion batteries,^9^ and thermoelectric conversion materials,^10^ taking advantages of its low reflectivity, large specific surface area and large space to accommodate volume expansion, and low thermal conductivity. Fabrication of high-aspect-ratio structures has been studied for the production of micro electro mechanical systems (MEMS),^11^ through-silicon via (TSV),^12,13^ and X-ray diffractive optics^14,15^ as well as for wafer dicing.^16^ Complex 3D structures, *e.g.* helical pores,^17,18^ spiraling pillars created by grid-shaped and star-shaped catalysts,^19^ nanocone arrays,^20^ and zigzag wires,^21^ have also been reported. We successfully enhanced the conversion efficiency of solar cells by using Si nanohole arrays.^6,22,23^ We also reported that adhesive metal film electrodes can be directly formed on Si substrates only through electroless processes by using the metal particles remained at the bottom of the pores as catalysts for the plating.^24^

**Fig. 1:**
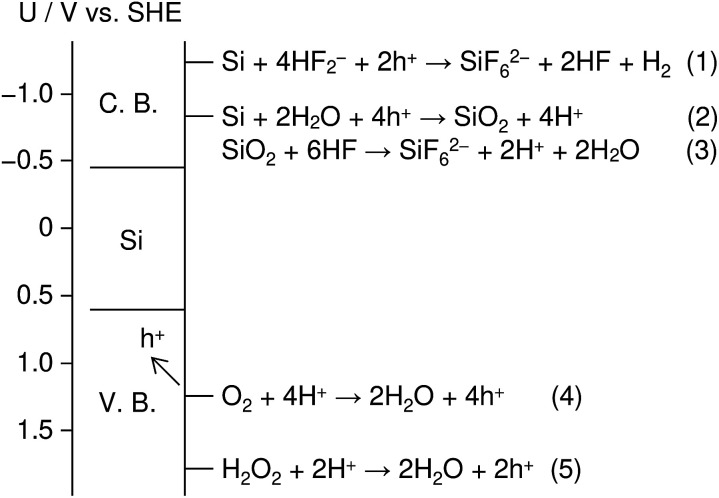
Energy band diagram of Si in a HF solution and standard electrode potentials of the reactions involved in metal-assisted etching. C. B. and V. B. indicate conduction band and valence band, respectively.

**Fig. 2:**
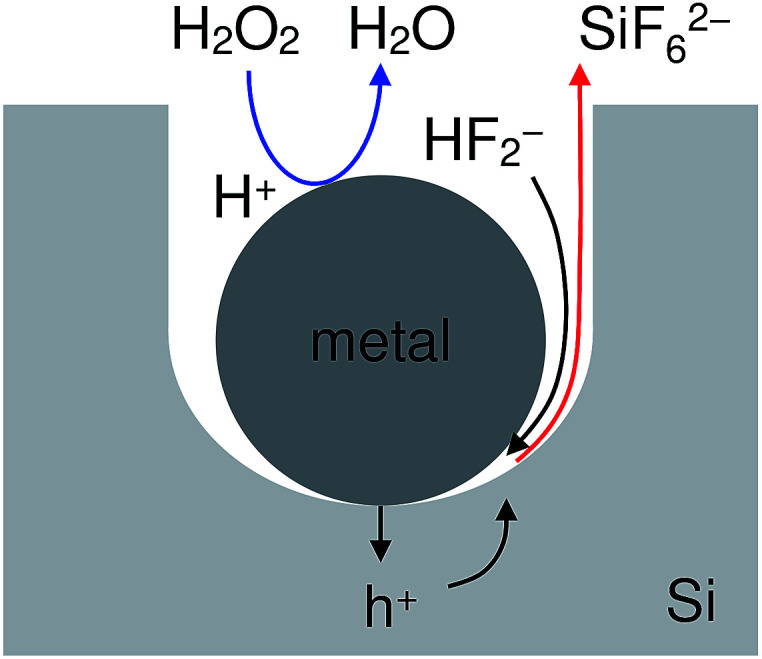
Schematic diagram of a model of the etching process.

Fig. 1 shows an energy band diagram of Si in a HF solution and standard electrode potentials of the reactions involved in the metal-assisted etching.^25,26^ Mechanism of the etching is explained by a local galvanic reaction, *i.e.* cathodic reduction of oxidizing agent (eqn (4) and (5) in Fig. 1) and anodic oxidation of Si (eqn (1) and (2) in Fig. 1). Positive holes are injected into the valence band of Si by the reduction of oxidizing agent. The positive holes are consumed for the direct dissolution of Si (eqn (1) in Fig. 1) or the formation of Si oxide by the reaction with water (eqn (2) in Fig. 1) followed by the dissolution by the fluoride species (eqn 3 in Fig. 1).^27^Fig. 2 shows one of the models of the etching process.^28–30^ The oxidizing agent is reduced on the metal catalysts and positive holes are injected into the metal/Si interface. Basically, the injected holes react with the fluoride species near the metal/Si interface, and hence, the Si surface under the metal catalysts is preferentially etched. The fluoride species and the reaction products are transported through the gap between the metal and Si. However, the mechanism of etching is still open to discuss.^1,2^

The etching behavior changes with various experimental parameters, *e.g.* etchant and Si wafer.^13,15,18,21,27,31–37^ Metal catalyst is also an important factor to change the porous structure.^25,26,38–41^ Lee at al.^38^ performed the etching of *p*-Si (100) in HF–H_2_O_2_ systems using silver (Ag), gold (Au), and platinum (Pt) particles, and they demonstrated that the etching behavior changes depending on the kind of metal as well as the shape of particles. We have found that the etching proceeds even without oxidizing agents when using palladium (Pd) catalysts which can catalyze the oxidation of Si.^25^ Asoh *et al.*^39^ fabricated macroporous Si using patterned Au and Pt–Pd films, and they found that straight pores were formed by the Au films while the Pt–Pd films causes an excessive pore widening with a high etching rate. However, systematic study of the effect of metal species on the etching behavior at a constant experimental condition is still limited.

We previously performed the etching of *n*-Si (100) in HF + O_2_ systems using Ag, Au, Pt, and rhodium (Rh) particles deposited with a constant metal coverage and evaluated the etching rate based on the mass loss of substrate.^26,41^ When the HF concentration was sufficiently low, the etching rate was determined by the HF concentration but not the kind of metal, indicating that the supply of fluoride species was rate-limiting step. When the HF concentration increased, the etching rate was higher in the order of the Rh-, Pt-, Au-, and Ag-deposited substrates. This order corresponded to that of the catalytic activity of the deposited materials for the reduction of dissolved oxygen. Not only the etching rate but also the morphological control is essential for microfabrication and various applications of nanostructured semiconductors. However, the morphology changes caused by the difference of the metal species have not been investigated in detail. In this work, *n*-Si (100) substrates modified with Ag, Au, and Pt particles were etched in a HF + H_2_O_2_ solution, and the porous structures were compared with the evaluation of the mass loss of substrate. In order to discuss the behavior of the positive holes injected from the metal particles, the etching was performed in the dark that avoids the generation of electron–hole pairs by photo excitation.

## 2. Experimental

A single-crystalline *n*-type Si wafer (CZ, (100), 0.5–10 Ω cm, 725 ± 25 μm) was cut into 3 × 3 cm^2^ squares. The Si substrate was ultrasonically cleaned in acetone for 5 min, etched in a CP4A solution (50% HF : 60% HNO_3_ : 99% CH_3_COOH : H_2_O = 3 : 5 : 3 : 22 in volume) for 3 min, and immersed in a 7.3 M (mol dm^−3^) HF aq. for 2 min in order to remove the oxide film. The Si substrate was rinsed with ultrapure water after each treatment, and dried at the end of the HF treatment by blowing nitrogen gas.

In this work, we modified the metal particles on the Si surface by electroless displacement deposition,^42^ which enables us to produce porous Si only through electroless wet processes. First, the back side of the Si substrate was covered with a masking tape. For the deposition of Ag, Au, or Pt particles, the Si substrate was immersed in a 1 mM AgNO_3_ + 0.15 M HF aq. at 278 K for 20 s, a 1 mM HAuCl_4_ + 0.15 M HF aq. at 278 K for 25 s, or a 3 mM K_2_PtCl_4_ + 0.15 M HF aq. at 313 K for 180 s, respectively. After the deposition, the Si substrate was rinsed with ultrapure water and dried with nitrogen gas. After removing the masking tape, four pieces of 1 × 1 cm^2^ squares were cut out from the central part of the 3 × 3 cm^2^ square in order to remove the edge of the Si substrate.

The metal-assisted etching was performed by immersing the metal-deposited Si substrate in a 0.08 M H_2_O_2_ + 6.6 M HF aq. at 298 K for 60 s in the dark. The volume of the etchant was 0.050 dm^3^. After the etching, the Si substrate was rinsed with ultrapure water and dried with nitrogen gas.

Surface and cross-sectional observation of the samples was performed using a scanning electron microscope (SEM) (JEOL Ltd, JSM-7001F). The mass of samples was measured before and after the etching using a microbalance (Mettler-Toledo International Inc., XP2U) for the evaluation of the mass loss by the etching.

## 3. Results

### 3.1. Microstructure of porous Si

**Fig. 3:**
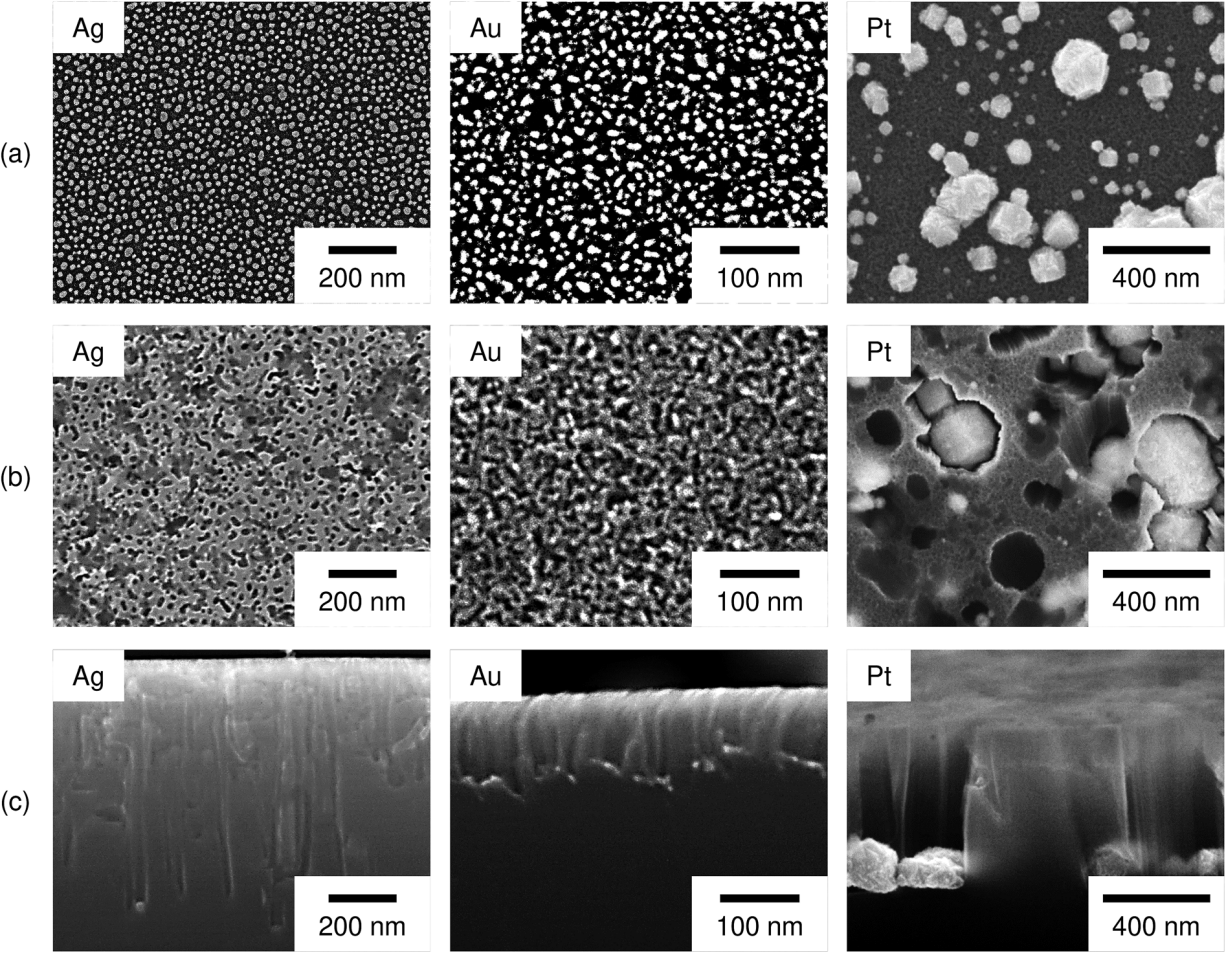
Surface SEM images of *n*-Si (100) substrates (a) after the deposition of Ag, Au, and Pt particles, (b) after the etching, and (c) the cross-sectional images after the etching. The etching was performed in a 0.08 M H_2_O_2_ + 6.6 M HF aq. at 298 K for 60 s in the dark.

Fig. 3 shows surface SEM images of the Si substrates after the deposition of Ag, Au, and Pt particles, after the etching, and the cross-sectional images after the etching. As shown in Fig. 3a, roughly spherical metal particles were separately deposited on the Si surface. The metal coverage on the Si surface became approximately constant (32 ± 3%, 30 ± 2%, and 29 ± 2% for the Ag, Au, and Pt particles, respectively) under the present deposition conditions. Then, we can assume that the catalyst area for the H_2_O_2_ reduction has not much difference among the Ag, Au, and Pt particles. The metal coverage was obtained as a percentage of white parts of the binary image of the metal-deposited Si surface. To determine the average coverages of the metal particles, we used multiple images captured at different positions and different magnifications, *i.e.* Ag: 12 images (4 images at 100 000× magnification, 4 images at 150 000× magnification, 4 images at 250 000× magnification), Au: 9 images (3 images at 150 000× magnification, 6 images at 300 000× magnification), and Pt: 5 images at 10 000× magnification. Note that the size of particles is inevitably different among the metals deposited with a constant coverage, since the nucleation behavior is different depending on the metal species.^42^

Fig. 3b shows the Si surface after the etching. We can see the pores whose size is similar to that of the metal particles. This indicates that the Si surface directly beneath the metal particles were selectively etched and the metal particles sunk into the Si substrate.

Fig. 3c shows the cross-section of the Si substrates after the etching. Straight pores were formed in the vertical direction. The metal particles were observed at the bottom of pores and the pore widening seems not to be significant. In some cases, we cannot observe the metal particles in the pores. This should be because the metal particles were removed when the Si substrate was cleaved or the etching direction was out of the straight. The depth of pores was considerably different depending on the metal species, *i.e.* it was larger in the order of Ag, Pt, and Au-deposited substrates (750 ± 80 nm, 630 ± 50 nm, and 104 ± 9 nm, respectively). Note that not only the vertical pores but also the non-vertical pores were observed. However, it was difficult to obtain the length of the non-vertical pores from the cross-sectional image. On the other hand, the etching proceeded even when the etching direction was out of the straight. Therefore, we approximately obtained the average depth of pores from the straight pores in which the starting point and the ending point of the etching were clearly observed with the metal particle at the bottom. To determine the average depth of pores formed by Ag, Au, and Pt particles, 44, 33, and 81 pores were measured, respectively.

**Fig. 4:**
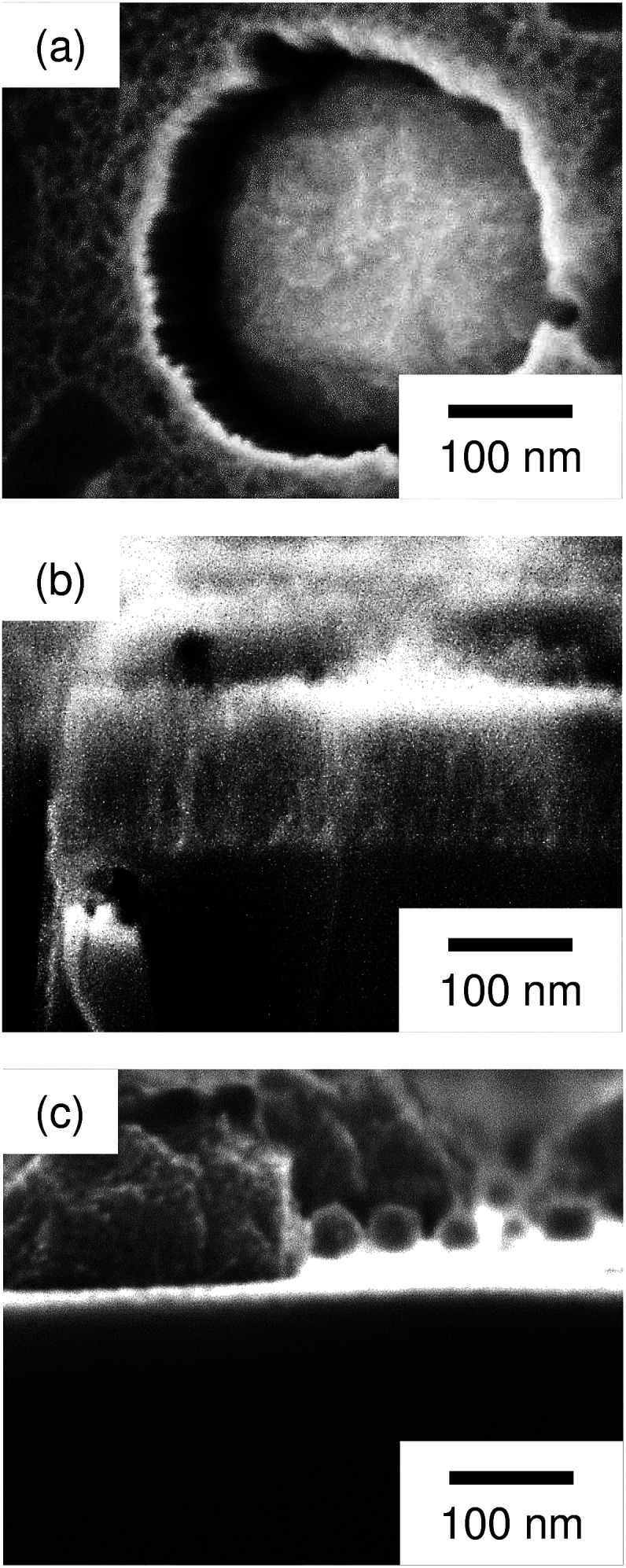
Enlarged images of (a) the surface and (b) cross section of the Pt-deposited substrate after the etching, and (c) the cross section before the etching.

Regarding the porous Si produced by the Pt particles, a layer whose brightness is different from that of bulk Si was observed at the top-surface of the Si substrate (see Fig. 3c). Fig. 4 shows enlarged images of the surface and cross section of the Pt-deposited substrate after the etching, and the cross section before the etching. In Fig. 4a, we can see randomly formed mesopores whose size is much smaller than the pores formed under the Pt particles. The mesoporous layer was observed over the entire surface of the Pt-deposited side of the Si substrate and its thickness was almost constant (as shown in Fig. 4b, the thickness was *ca.* 120 nm). Such a thick layer was not observed before the etching (see Fig. 4c), although the Si surface is dissolved during the metal deposition process.^42^ It should also be noted that the etching of the back side of the Si substrate was not observed.

### 3.2. Mass loss by the etching

**Fig. 5:**
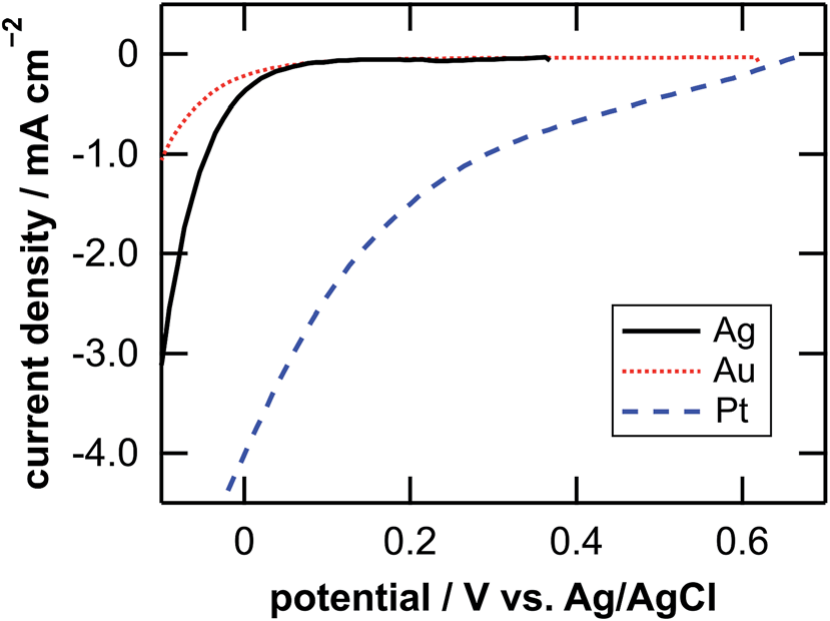
Current density *versus* potential curves of Ag (solid line), Au (dotted line), and Pt (dashed line) wires in the etchant measured from the open circuit potentials to the negative direction at a sweep rate of 20 mV s^−1^.

Table 1 lists the coverage of metal particles, mass loss of substrate, depth of pores observed by SEM, calculated depth of pores, and calculated depth of general corrosion. The calculation of the depth of the pores and the general corrosion is described later (Section 4.1). The mass loss was larger in the order of Pt-, Ag-, and Au-deposited substrates. This is explained by the catalytic activity of the metals for the local cathodic reaction of H_2_O_2_ reduction. Fig. 5 shows current density *versus* potential curves of metal wires in the etchant measured from the open circuit potentials to the negative direction at the sweep rate of 20 mV s^−1^. The on-set potential of the cathodic current was more positive in the order of Pt, Ag, and Au electrodes. This indicates that the overpotential for the H_2_O_2_ reduction is less in the same order, which corresponds to that the mass loss is larger in the order of Pt-, Ag-, and Au-deposited substrates. The current density of the local galvanic reaction, namely the etching rate, is considered to be higher when the overpotential is lower.^26^ Note that the composition change of the etchant is negligible. If we assume that the mass loss of the Pt-deposited substrate (75.9 μg, Table 1) is due to the reactions of eqn (2), (3) and (5) in Fig. 1, 0.13% of H_2_O_2_ and 0.0050% of HF are consumed in the 0.050 dm^3^ of the 0.08 M H_2_O_2_ + 6.6 M HF aq.

**Table 1:** Metal coverage, mass loss of substrate, observed depth of pores, calculated depth of pores, and calculated depth of general corrosion. The etching was performed in a 0.08 M H_2_O_2_ + 6.6 M HF aq. at 298 K for 60 s in the dark. Pt-assisted etching was additionally performed by changing the H_2_O_2_ concentration to 0.04 M

Metal	Coverage %	Mass loss Δ*m*/μg	Depth of pores		Depth of corrosion
			*d*_obs_/nm	*d*_cal_ a/nm	*d*_corr,cal_ a/nm
Ag	32 ± 3	57.5	750 ± 80	770 ± 60	7 ± 30
Au	30 ± 2	19.3	104 ± 9	280 ± 20	52 ± 7
Pt	29 ± 2	75.9	630 ± 50	1100 ± 70	140 ± 30
Pt^b^	29 ± 2	41.8	310 ± 30	610 ± 40	90 ± 10

a The calculation method is described in the Section 4.1.

b [H_2_O_2_] = 0.04 M.

## 4. Discussion

### 4.1. General corrosion

**Fig. 6:**
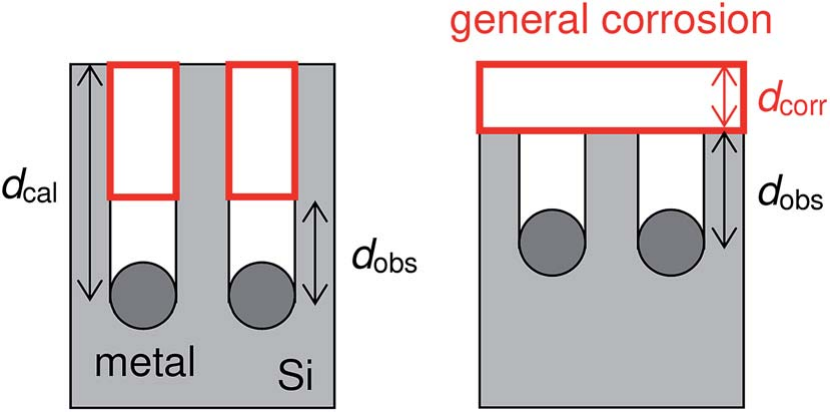
Schematic diagrams of the calculated depth of pores and the general corrosion.

It is interesting that the depth of pores formed by the Pt particles was smaller than that formed by the Ag particles even though the mass loss of the Pt-deposited substrate was larger than that of the Ag-deposited substrate (see Table 1). Here, we calculate the depth of pores using the mass loss of substrate. Assuming that the mass loss is only due to the formation of straight pores caused by the dissolution of Si directly beneath the metal particles, the depth of pores can be obtained by the following equation:6
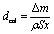
where *d*_cal_ is the calculated depth of pores, Δ*m* is the mass loss of substrate, *ρ* is the density of Si (2.3296 g cm^−3^), *S* is the surface area of Si (1.0 cm^2^) on which the metal particles were deposited, and *x* is the average coverage of the metal particles. As shown in Table 1, in the case of the Ag-deposited substrate, the calculated depth of pores was almost the same as the observed depth. On the other hand, in the cases of the Au and Pt-deposited substrates, the calculated depth of pores was much larger than the observed depth. This suggests that the mass loss is not only due to the formation of straight pores but also due to the dissolution of the top-surface of the Si substrate, which is general corrosion. Fig. 6 shows schematic diagrams of the calculated depth of pores and the general corrosion. Assuming that the volume of the general corrosion corresponds to that involved with the overestimated depth of pores, we can calculate the depth of general corrosion using the following equation:

7 *Sx*(*d*_cal_ − *d*_obs_) = *Sd*_corr_

where *d*_corr_ is the depth of general corrosion and *d*_obs_ is the depth of straight pores observed by the SEM. The general corrosion was more significant in the order of the Pt-, Au-, and Ag-deposited substrates (140 ± 30 nm, 52 ± 7 nm, and 7 ± 30 nm, respectively). We conclude that the metal species make a difference to the etching of the Si surface under the metal particles as well as between them.

In order to discuss a possible reason of the general corrosion, we confirmed the photoluminescence of the sample etched by the Pt particles. Under the illumination of a black light (Nihon Kagaku Engineering Co. Ltd, NV-102), visible (orange) luminescence was observed by naked eyes. This indicates the presence of nanometer-sized pores.^43^ It has been known that nanometer-sized Si is chemically dissolved in HF solutions.^44^ Thus, the mesoporous layer formed with the metal-assisted etching can be dissolved in the etchant during the etching process. The general corrosion can occur due to the dissolution of the mesoporous layer.

Note that the calculation of the general corrosion above does not include the porosity of the mesoporous layer, which should be one of the reasons of the mass loss of the Pt-deposited substrate. Assuming that the mesoporous layer with a constant thickness is formed on the top-surface of the Si substrate which is not etched by the Pt particles, the mass loss of substrate can be expressed as follows:

8 Δ*m* = *d*_obs_*ρSx* + *t*_meso_*p*_meso_*ρS*(1 − *x*)+*d*_corr_*ρS*

where *t*_meso_ and *p*_meso_ are the thickness and porosity of the mesoporous layer, respectively. If we roughly assume a 120 nm thick mesoporous layer with a porosity of 0.5, the depth of general corrosion is estimated to be *ca.* 100 nm by using eqn (8).

### 4.2. Formation of mesoporous layer

Formation of mesoporous Si by the metal-assisted etching has been known so far^1–5,7,28,31,32,34,37,40,45,46^ as well as pore widening^36,39,41^ and pit formation at the location away from the catalysts.^47,48^ Such phenomena have been usually observed when using the catalysts with a high catalytic activity for the reduction of oxidizing agents. On the other hand, Chartier *et al.*^31^ performed the etching with Ag particles in the etchants in which the HF/H_2_O_2_ ratio was systematically changed. They demonstrated that mesoporous Si was formed with decreasing the HF/H_2_O_2_ ratio. In our case, the molar ratio [HF]/([HF]+[H_2_O_2_]) was 0.99. According to their report, mesoporous Si is not observed in this condition. They also observed cone-shaped pores, craters, and the polishing when the HF concentration is sufficiently low. Lianto *et al.*^47^ performed the etching with patterned Au strips and demonstrated that the pit formation became significant with increasing the H_2_O_2_ concentration. We observed the pore widening with Rh and Pt particles in HF–O_2_ systems at low HF concentrations.^41^ Recently, Akan *et al.*^15^ demonstrated, for the fabrication of X-ray zone plates, that the surface roughness became significant and the etching depth under Au catalysts became smaller when the H_2_O_2_ concentration is high. These results suggest that positive holes are consumed for the Si dissolution at the location away from the catalysts when the supply of fluoric species to the metal/Si interface is insufficient relative to the hole injection associated with the H_2_O_2_ reduction.

**Fig. 7:**
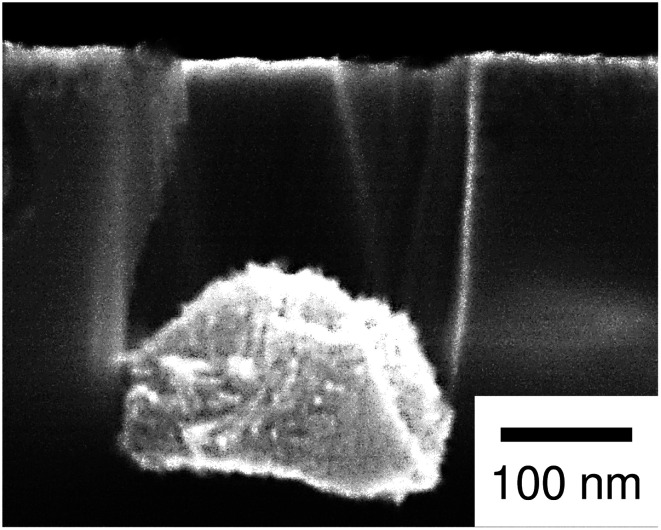
Enlarged image of the cross section of the Pt-deposited substrate after the etching with the low concentration of H_2_O_2_ (0.04 M).

According to the discussion above, it is reasonable that the general corrosion was significant when using the Pt particles with a high catalytic activity for the H_2_O_2_ reduction. On the other hand, we additionally performed the etching with the Pt particles in an etchant with a low H_2_O_2_ concentration of 0.04 M (see Table 1). In this condition, the mass loss of substrate was smaller than that of the Ag-deposited substrate, while the calculated depth of pores was much smaller than the observed depth of pores (25 pores were evaluated to determine the average depth). We also observed the mesoporous layer on the top-surface of the Si substrate (see Fig. 7). This implies that the ratio of the positive holes consumed for the straight pore formation and that consumed for the general corrosion is changed not only by the amount of injected holes but also by the kind of metal, although the mass loss of substrate does not correspond directly to the amount of injected holes (two-electron and four-electron reactions can be considered for the Si oxidization (eqn (1) and (2) in Fig. 1) and the mesoporous layer can be chemically dissolved).

Recently, band-bending at the metal/Si interface and polarization of the Si substrate has been considered as important factors in the metal-assisted etching.^36,40,46,49–53^ For example, Kolasinski^51,53^ proposed the mechanism of the metal-assisted etching with the calculation of the band structures at the metal/Si interface formed by different kind of metals. He demonstrated that the band structure is favorable for the hole injection in the cases of Au and Pt with large work functions but not in the case of Ag with relatively small work function. Torralba *et al.*^46^ simulated the band modulation at a *p*-Si surface in the vicinity of Pt particles in an electrolyte. They demonstrated that the positive holes are injected into the bulk Si due to the ohmic nature at the Pt/Si interface, which induces an anodic polarization of the Si substrate and the formation of mesoporous layer at the Si surface away from the Pt particles. In our case, an *n*-Si (positive holes are minority carriers) was etched in the dark (electron–hole pairs are not generated by photo excitation). Thus, the injected holes are considered to be diffused into the bulk Si that induces the anodic polarization as well as into the top-surface of the Si substrate for the mesopore formation. The fact that the etching of the back side of the Si substrate was not observed is explained by that the thickness of the Si wafer (725 ± 25 μm) is larger than the diffusion length of the positive holes (200–500 μm). The mesopore formation and the general corrosion should occur at the location to which the injected holes can diffuse. Considering the large work function of Au, the mesoporous layer can also be formed in the case of the Au-deposited substrate, but not observed in our case. In the present conditions, the mesoporous layer may be thin, making it difficult to clearly observe. For the detailed discussion, it will be necessary to investigate the band structure and the electrode potential caused by the different kind of metal particles. We also should consider the size of particles and characteristics of metals, *e.g.* Ag can be dissolved in the etchant^54^ and Au–Si alloy can be formed at the Au/Si interface.^55^

## 5. Conclusion

In this work, we investigated the metal-assisted etching of *n*-Si in a HF–H_2_O_2_ system in the dark using Ag, Au, and Pt particles electrolessly deposited at a constant coverage. The porous structure was considerably different depending on the metal species. The depth of straight pores formed directly beneath the metal particles was larger in the order of the Ag-, Pt-, and Au-deposited substrates. By considering the mass loss of substrate, we found that the top-surface of the Si substrate was dissolved during the etching even under the dark condition. The general corrosion was significant in the cases of the Au- and Pt-deposited substrates but not in the case of the Ag-deposited substrate. When we used the Pt particles as catalysts, mesoporous layer was clearly observed around the Pt particles. The general corrosion is explained by the formation and dissolution of the mesoporous layer. To the best of our knowledge, this paper discussed the general corrosion during the metal-assisted etching on the basis of the comparison of the mass loss of substrate with the depth of pores for the first time. We conclude that the kind of metal affects the etching of not only the Si surface under the metal particles but also the top-surface away from the metal particles. This should be carefully considered for the fabrication of Si nanostructures by the metal-assisted etching. Further investigations will be needed to understand the general corrosion during the metal-assisted etching.

## Conflicts of interest

There are no conflicts to declare.

## Supplementary Material
